# The effects of citrulline supplementation on meta-inflammation and insulin sensitivity in type 2 diabetes: a randomized, double-blind, placebo-controlled trial

**DOI:** 10.1186/s13098-021-00669-w

**Published:** 2021-05-05

**Authors:** Fatemeh Abbaszadeh, Samaneh Azizi, Majid Mobasseri, Mehrangiz Ebrahimi-Mameghani

**Affiliations:** 1 Student Research Committee, School of Nutrition and Food Sciences, Tabriz University of Medical Sciences, Tabriz, Iran; 2 Endocrine Research Center, School of Medicine, Tabriz University of Medical Sciences, Tabriz, Iran; 3 Nutrition Research Center, Department of Biochemistry and Diet Therapy, School of Nutrition and Food Sciences, Tabriz University of Medical Sciences, Tabriz, Iran

**Keywords:** Citrulline, Diabetes mellitus, type 2, Meta-inflammation, Insulin sensitivity

## Abstract

**Background:**

This study aimed to examine the effects of l-citrulline (*l-CIT*) on low-grade inflammation (meta-inflammation) and insulin sensitivity in type 2 diabetes (*T2D*) patients since it has exhibited hypoglycemic and anti-inflammatory effects in most animal studies.

**Methods:**

In this double-blind, placebo-controlled randomized clinical trial, 54 patients with T2D referred to specialized clinics of Tabriz University of Medical Sciences were assigned to l-CIT group (receiving orally one 3 g sachet of l-CIT daily before breakfast) or placebo group (receiving orally one 3 g sachet of microcrystalline cellulose daily before breakfast) for eight weeks. Serum levels of fasting blood glucose, hemoglobin A1c (*HbA1c*), CIT, monocyte chemoattractant protein 1 (*MCP-1*), interleukin-6 (*IL-6*), and toll-like receptor 4 (*TLR-4*) were determined. The quantitative insulin sensitivity check index (*QUICKI*) and homeostatic model assessment of β-cell function (*HOMA-B*) index were estimated at the baseline and post-intervention.

**Results:**

No significant difference was observed between the studied parameters at the baseline. l-CIT supplementation significantly reduced not only serum concentrations of fasting blood glucose but also HbA1c, serum IL-6 and TLR-4 levels in the l-CIT group (p < 0.05). Additionally, at the end of the study serum levels of CIT increased significantly in l-CIT group compared to the baseline and placebo group. Fasting blood glucose concentrations and HbA1c significantly decreased after the intervention compared to the placebo. There was no significant difference in serum IL-6, TLR-4, MCP-1 levels, as well as QUICKI and HOMA-B index between the two groups, even after adjusting for baseline variables and confounders.

**Conclusions:**

Our findings revealed that, although l-CIT supplementation significantly reduced fasting blood glucose concentrations, HbA1c and increased serum levels of CIT. It seems it could not significantly improve insulin sensitivity and meta-inflammation biomarkers. Additional studies with longer duration and different doses of l-CIT are required.

*Trial registration* The protocol of this clinical trial is registered at the Iranian Registry of Clinical Trials (registration no: IRCT20100209003320N16 at www.irct.ir)

## Background

Diabetes mellitus is a prevalent metabolic disorder which results in the abnormal function and insulin secretion [1]. In 2019, International Diabetes Federation (IDF) estimated that 463 million individuals (9.3%) suffer from diabetes around the world which is expected to reach 700 million (10.9%) by 2045 [2]. The type 2 diabetes (T2D) accounts for 90 to 95% from all diabetic patients [3], which causes by β-cell dysfunction and insulin resistance (IR) [4, 5]. In addition to the IR, meta-inflammation—a low-grade chronic metabolic inflammation—is crucial to T2D pathogenesis [6]. In diabetes, chronic hyperglycemia and elevated free fatty acid (FFA) levels resulted in the IR and meta-inflammation [7]. In fact, the elevated FFA levels result in the IR by mitigating the phosphorylation of insulin receptor substrate-1 (IRS-1), stimulated by insulin, and its related phosphoinositide 3-kinase (PI3-K) activity [7, 8]. Conversely, IR in fat tissue can itself increase the circulating levels of FFAs by increasing the lipolysis (defective cycle) [9].

FFAs released from adipocyte play a critical role in the meta-inflammation by toll-like receptors (TLRs), which is one of the main classes of pattern-recognition receptors (PRRs) in the mammalian cells [10]. Toll-like receptor-4 (TLR4) (the most important type of TLRs in diabetes) induces the activation of meta-inflammation mediators, referred to the nuclear factor kappa B (NF-ҠB) signaling pathway [11]. Through the activated NF-ҠB pathway, the expression of some inflammatory genes (e.g., IL-1β, IL-6, and IL-8) and MCP-1 increase which leads to IR [12, 13]. These conditions, i.e., hyperglycemia, IR, and meta-inflammation, cause several macrovascular and microvascular complications in the T2D patients [14]. Moreover, using anti-inflammatory agents may be therapeutically useful to reduce the IR and/or improving β-cell function which may delay the complications of diabetes.

l-CIT—an α-amino acid compound existing in the high concentrations in watermelon—is a vital part of urea cycle in kidneys and liver [15, 16]. Endogenous l-CIT is produced from glutamine by the enterocytes [15]. Then, l-CIT is taken up by kidneys for the de novo synthesis of arginine [17]. Therefore, l-CIT is an endogenous precursor of l-arginine and nitric oxide (NO) [18, 19]. NO plays a significant role in the health maintenance and artery vasodilation [20]. Furthermore, it seems to be crucial to regulate the metabolism of systemic glucose and insulin delivery to peripheral tissues [21]. Furthermore, decreased bioavailability of NO was reported during the diabetes [22, 23]. Earlier animal studies indicated that the treatment with l-CIT or watermelon components reduces the meta-inflammation biomarkers such as MCP-1, TLR-4, and IL-6, which may have a hypoglycemic effect [24–27]. Then, it was speculated that l-CIT supplementation would ameliorate the risk factors of the T2D by enhancing the glucose homeostasis and attenuating inflammation. Therefore, this study aimed to examine the effects of l-CIT supplementation on glucose homeostasis and meta-inflammation in the T2D patients.

## Methods and materials

### Subjects

Patients with T2D receiving metformin and sulfonylureas, aged 25–55 years with hemoglobin A1c (HbA1c) < 9% and body mass index (BMI) between 25–35 kg/m^2^ were selected. Exclusion criteria were smoking, pregnancy, lactation, and post-menopause, those suffering from allergy, cancer, cardiovascular, inflammatory, intestinal, kidney, liver, and malabsorption diseases, hyperthyroidism/hypothyroidism, and polycystic ovary syndrome (PCOS) and followed weight-loss diets, receiving insulin, Sodium–glucose cotransporter-2 (SGLT2) inhibitors, dipeptidyl peptidase-4 (DPP-4) inhibitors and glucagon-like peptide 1 (GLP-1) receptor analogues, nonsteroidal anti-inflammatory medications (NSAIDs), or any medication for lowering blood pressure, lipid, interacting with NO or any nutritional supplements during the last three months. One week before the intervention, participants were requested to abstain from eating watermelon and high l-CIT foods and drinking watermelon juice until the end of the intervention.

Due to the reported mean (± SD) of glucose by Hickner et al. [28], as well as a 95% confidence level and a power of 80%, the sample size was calculated to be 23 for each group which increased to 27 by considering a 20% dropout. Furthermore, 54 patients referred to specialized and subspecialized clinics of Tabriz University of Medical Sciences were randomly allocated into "*l*-*CIT group*" (n = 23) or "*placebo group*" (n = 22). All patients were asked to sign an informed consent form after a full explanation of study procedures, and dietary recommendation was given for diabetic patients at the beginning of study.

### Experimental design

This double-blind placebo-controlled randomized clinical trial was performed from July to October 2019. The study was approved by the ethics committee of research vice-chancellor of Tabriz University of Medical Sciences, Tabriz, Iran (Ethics code: IR.TBZMED. REC. 1398.739); the study was registered in the Iranian Registry of Clinical Trials (IRCT20100209003320N16 at www.irct.ir).

Fifty-four T2D patients were randomly allocated to "*placebo group*" (n = 27) (receiving a single sachet of microcrystalline cellulose (3 g/days, Linyi Jindi Chemical Co., Ltd., China) or "*l-CIT group*" (n = 27) (receiving a single sachet of l-CIT (3 g/days, Bulk Supplements Co., Ltd., the USA) dissolved in a glass of water before breakfast every day for eight weeks. The dose of l-CIT was nearly the same as doses in the previous clinical trials which has been reported safe and effective [29, 30]. This random allocation was conducted using a random block using Random Allocation Software (RAS) version.1.0 (M. Saghaei, Department of Anesthesia, Isfahan University of Medical Sciences (IUMS), Isfahan, Iran) and an independent third party who did not participate in the clinical part of research. The clinicians, patients, and raters had no information regarding the groups.

### Anthropometric and dietary assessments and blood pressure

Height and weight were measured using a calibrated stadiometer (Seca, Hamburg, Germany) and, consequently, BMI was estimated. Dietary intakes were assessed using a 3-day food diary. The subjects were asked to record their dietary intakes on 3 non-consecutive days (2 days and a weekend). Food diaries were completed based on the estimated values in household measurement and portion sizes. All reported portion sizes and amounts were converted to grams. Data on food intake were analyzed by Nutritionist-IV software (First Databank, Division, San Bruno, CA, USA) modified for Iranian foods. Blood pressure was measured with a sphygmomanometer twice 30 min apart in the seated position after 5 min of rest each time.

### Laboratory analysis

Blood samples were taken after 12–14 h of fasting which were immediately centrifuged. The serum level of fasting blood glucose was measured on the same day, while the rest of serum was stored at − 80 °C until analysis. Commercial kits were used to assess serum concentrations of fasting blood glucose (Pars-Azmoon Co., Tehran, Iran), insulin (Monobind, USA) and serum levels of CIT (the Bioassay Technology Laboratory Co, Ltd, Yangpu District, Shanghai, China) based on the enzyme-linked immunosorbent assay (shortened as, ELISA) technique. HbA1c was measured by photometry in whole blood using Pars Azmoun Company kit (Pars Azmoun, Iran) and Hitachi autoanalyzer (Hitachi-917, Tokyo, Japan). Serum IL-6, TLR-4, MCP-1 concentrations were determined using a high sensitivity Enzyme-linked immunosorbent assay (ELISA) kit (crystal day, Shanghai, China). Finally, insulin sensitivity estimates were calculated using QUICKI [31] and HOMA-B [32] index for estimating β-cell function as follows: QUICKI score = 1/(log(fasting insulin [μU/mL]) + log(fasting glucose [mg/dL])) HOMA-B = (360 × fasting insulin [µIU/mL])/(fasting glucose [mg/dL] − 63).

### Statistical analysis

The SPSS software Ver. 26 (IBM Corp., Armonk, NY, USA) and STATA software Ver. 15 (StataCorp, College Station, TX, USA) was applied for data analysis, and data were analyzed by the per-protocol method. Kolmogorov–Smirnov test was carried out to investigate data distribution, and data for normal and non-normal distributed quantitative variables were presented as mean and standard deviation (Mean ± SD) or median in percentile ranges (25th and 75th percentiles), respectively. Within-group changes over the intervention were examined using paired samples t-test and Wilcoxon signed-rank test. In contrast, the independent samples t-test and Mann–Whitney U test were applied to explore between-groups changes before and after the intervention. Furthermore, inter-group differences were analyzed using quantile regression and analysis of covariance (ANCOVA) tests by adjusting for the confounders. Chi-square and sign tests were conducted to examine between- and within-group differences between qualitative variables. p < 0.05 was considered to be a level of statistical significance.

## Results

### Baseline characteristics of trial patients

Among 54 patients who participated, 45 patients completed the trial (l-CIT group, n = 23; placebo group, n = 22). Nine of these patients dropped out. Four patients from l-CIT group: two persons because of the discontinued intervention, one person for not taking the supplement due to the plan and one person because of insulin consumption, as well as five patients from the placebo group: Three persons because of the discontinued intervention and two patients for not taking supplements as planned. Therefore, the data were analyzed for 45 patients. The patients reported no missing data (or values) or side effects through the intervention. Table 1 presents the baseline characteristics of the T2D patients. There were no differences in gender, age, antihyperglycemic medications (metformin or glibenclamide), and duration of diabetes among the groups (p > 0.05).

**Table 1:** Baseline characteristics of T2D patients

Characteristics	CIT (n = 23)	Placebo (n = 22)	p
	Mean ± SD	Mean ± SD	
Age (year)	47.57 (5.60)	49.86 (4.88)	0.151*
Duration of diabetes	4.26 (1.25)	4 (1.41)	0.515*
Gender	N (%)	N (%)	
Male	14 (60.90)	15 (68.20)	
Female	9 (39.10)	7 (31.80)	0.612**
Antihyperglycemic medications			
Metformin	6 (26.10)	5 (22.70)	
Sulfonylureas	7 (30.40)	3 (13.60)	0.349**
Both	10 (43.50)	14 (63.60)	

^*^ p based on Independent-samples t-test

^**^ p based on chi-squared test

### Dietary intake, anthropometric indices, and blood pressure

Following the supplementation, no significant differences were found not only in the dietary intakes of macronutrients, but also in weight and BMI at baseline between CIT and placebo groups. Systolic and diastolic blood pressure (SBP, DBP) decreased in the CIT group (p < 0·05); although, after adjusting for baseline values, no significant changes were observed among the groups (p > 0.05) (Table 2).

**Table 2:** Dietary intake, anthropometric indices, and blood pressure

Variables	CIT (n = 23)	Placebo (n = 22)	MD (95% CI)	p
Energy (Kcal/days)				
Baseline	2346.43 (462.87)	2260.09 (535.41)	86.343 (− 214.13, 386.82)	0.565^b^
End	2325.00 (367.42)	2194.68 (517.78)	68.89 (− 97.03, 234.81)	0.407^c^
MD (95% CI)	− 21.43 (− 153.91, 111.04)	− 65.40 (− 202.12, 71.30)		
p	0.740^a^	0.331^a^		
Energy from protein (%)				
Baseline	13.39 (1.03)	13.54 (1.79)	− 0.15 (− 1.02, 0.72)	0.724^b^
End	12.91 (0.90)	13.00 (1.82)	− 0.04 (− 0.88, 0.79)	0.911^c^
MD (95% CI)	− 0.47 (− 1.04, 0.08)	− 0.54 (− 1.48, 0.38)		
p	0.094^a^	0.239^a^		
Energy from carbohydrate (%)				
Baseline	55.65 (7.23)	57.59 (6.98)	− 1.93 (− 6.21, 2.34)	0.366^b^
End	57.17 (6.61)	56.31 (6.38)	1.86 (− 1.42, 5.15)	0.259^c^
MD (95% CI)	1.52 (− 1.48, 4.53)	− 1.27 (− 3.77, 1.22)		
p	0.306^a^	0.301^a^		
Energy from fat (%)				
Baseline	30.86 (7.33)	28.81 (6.98)	2.05 (− 2.25, 6.36)	0.342^b^
End	29.86 (6.60)	30.45 (5.59)	− 1.59 (− 4.68, 1.49)	0.303^c^
MD (95% CI)	− 1.00 (− 3.95, 1.95)	1.63 (− 0.77, 4.05)		
p	0.490^a^	0.173^a^		
Weight (kg)				
Baseline	84.00 (13.16)	78.97 (10.09)	5.02 (− 2.04, 12.10)	0.159^b^
End	84.19 (13.04)	78.97 (10.07)	0.23 (− 0.26, 0.73)	0.355^c^
MD (95% CI)	0.19 (− 0.25, 0.64)	0.00 (− 0.19, 0.20)		
p	0.388^a^	0.963^a^		
BMI (kg/m^2^)				
Baseline	29.77 (3.20)	28.26 (2.14)	1.51 (− 0.13, 3.15)	0.071^b^
End	29.84 (3.15)	28.25 (2.16)	0.09 (− 0.08, 0.27)	0.301^c^
MD (95% CI)	0.07 (− 0.09, 0.22)	− 0.01 (− 0.07, 0.06)		
p	0.389^a^	0.924^a^		
SBP (mmHg)				
Baseline	132.60 (11.16)	130.68 (13.56)	1.92 (− 5.52, 9.38)	0.605^b^
End	126.73 (11.14)	126.59 (13.74)	− 0.98 (− 7.16, 5.19)	0.749^c^
MD (95% CI)	− 5.87 (0.91, 10.81)	− 4.09 (− 0.87, 9.05)		
p	**0.022**^a^	0.101^a^		
DBP (mmHg)				
Baseline	92.43 (6.38)	90.40 (11.96)	2.02 (− 3.70, 7.75)	0.480^b^
End	88.26 (8.47)	87.27 (11.20)	0.30 (− 5.43, 6.03)	0.916^c^
MD (95% CI)	− 4.17 (0.41, 7.93)	− 3.13 (− 2.83, 9.10)		
p	**0.031**^a^	0.287^a^		

Bold values denote statistical significance at the* p* < 0.05 level

*BMI* body mass index, *SBP* systolic blood pressure, *DBP* diastolic blood pressure

Mean (SD) and Mean difference (95% CI) are presented for normally distributed data, median (25th and 75th percentiles) are presented for data not normally distributed. p < 0.05 was considered significant

^a^p based on Paired samples t-test. Data are presented as the mean difference (95% CI)

^b^p based on Independent samples t-test. Data are presented as the mean difference (95% CI)

^c^p based on ANCOVA adjusted for baseline values. Data are presented as the mean difference (95% CI)

### Glycemic response, serum levels of CIT and inflammatory factors

Table 3 shows the serum levels of CIT, glycemic response, and meta-inflammatory biomarkers before and after the trial in both groups. In the CIT group, the serum concentrations of fasting blood glucose and HbA1c were significantly reduced with respect to baseline and the placebo group, while the serum concentrations of TLR4 and IL-6, significantly reduced compared to baseline (p = 0.002, and p < 0.001, respectively). At the same time, an increase in serum IL-6 concentration and HbA1c was found in the placebo group (p = 0.042, p = 0.024, respectively). In fact, serum insulin and HOMA-IR did not change significantly over the intervention in both groups (data are not shown).The group analysis at the end of study for the changes in the serum levels of CIT, glucose response, and meta-inflammatory biomarkers indicated the significant differences in the serum levels of CIT as well as fasting blood glucose, insulin, HOMA-IR, and HbA1c after adjusting for the baseline values, diabetes duration, and changes in energy intake and BMI (p = 0.006, p = 0.032, p = 0.025, p = 0.037, p = 0.001, respectively). No statistically significant effects of l-CIT supplementation were observed for the insulin sensitivity indices (HOMA-B and QUICKI), as well as meta-inflammatory biomarkers.

**Table 3:** Changes in serum levels of CIT, glycemic response and inflammatory factors over the study

Variables	CIT (n = 23)	Placebo (n = 22)	MD (95% CI)	p
Citrulline (nmol/mL)				
Baseline	13.38 (11.74, 44.52)	11.01 (3.62, 72.07)	2.37	0.318^e^
End	30.47 (20.91, 68.76)	7.09 (3.27, 55.45)	0.79 (0.24, 1.35)	**0.006**^**f**^
MD (95% CI)	17.09	− 3.92		
p	**< 0.001**^**d**^	0.168^d^		
Fasting blood glucose (mg/dL)				
Baseline	157.91 (41.73)	163.40 (59.76)	− 5.49 (− 36.36, 25.37)	0.721^b^
End	134.91 (32.23)	160.72 (61.44)	− 21.64 (− 41.32, − 1.96)	**0.032**^c^
MD (95% CI)	− 23 (− 33.52, − 12.47)	− 2.68 (− 21.01, 15.64)		
p	**< 0.001**^a^	0.764^a^		
HbA1c (%)				
Baseline	7.21 (1.21)	6.97 (1.53)	0.24 (− 0.59, 1.07)	0.562^b^
End	6.75 (1.35)	7.28 (1.31)	− 0.66 (− 1.02, − 0.307)	**0.001**^**c**^
MD (95% CI)	− 0.46 (− 0.74, − 0.17)	0.31 (0.04, 0.58)		
p	**0.003**^a^	**0.024**^**a**^		
HOMA-B				
Baseline	66.77 (39.46, 109.87)	55.93 (25.49, 112.40)	10.84	(0.555)^e^
End	68.05 (28.37, 124.22)	79.52 (35.54, 152.95)	− 0.08 (− 0.22, 0.05)	**0.238**^**f**^
MD (95% CI)	1.28	23.59		
p	0.808^d^	0.277^**d**^		
QUICKI				
Baseline	0.28 (0.27, 0.31)	0.29 (0.27, 0.36)	− 0.01	(0.874)^e^
End	0.30 (0.28, 0.34)	0.29 (0.27, 0.30)	134.96 (− 81.30, 351.24)	0.214^f^
MD (95% CI)	0.02	0.00		
p	0.211^d^	0.140^d^		
MCP-1(ng/l)				
Baseline	67.02 (56.04, 593.27)	102.38 (59.22, 798.10)	− 35.36	(0.570)^e^
End	60.30 (43.37, 686.51)	95.38 (50.79, 916.19)	0.02 (− 0.16, 0.22)	0.757^f^
MD (95% CI)	− 6.72	− 7.00		
p	0.094^d^	0.338^d^		
TLR4)ng/ml)				
Baseline	4.98 (4.41, 19.11)	7.79 (4.73, 18.38)	− 2.81	(0.555)^e^
End	4.31 (4.07, 18.17)	7.29 (4.63, 18.94)	− 6.45 (− 13.89, 0.98)	0.087^f^
MD (95% CI)	− 0.67	0.50		
p	**0.002**^d^	0.846^d^		
IL-6 (ng/l)				
Baseline	94.40 (61.38, 592.51)	90.78 (59.65, 269.62)	3.62	(0.794)^e^
End	88.62 (50.77, 480.35)	97.20 (70.60, 386.25)	− 0.08 (− 0.18, 0.01)	0.109^f^
MD (95% CI)	− 5.78	6.42		
p	** < 0.001**^d^	**0.042**^d^		

Bold values denote statistical significance at the* p* < 0.05 level

*HbA1c* Glycated hemoglobin, *HOMA-B* homeostatic model assessment for beta-cell function, *QUICKI* quantitative insulin sensitivity check index, *MCP-1* monocyte chemoattractant protein-1, *TLR-4* toll-like receptor 4, *IL-6* interleukin 6

Mean (SD), and mean difference (95% CI) are presented for normally distributed data; median (25th and 75th percentiles), median difference and coefficient (95% CI) are presented for data not normally distributed

^a^p based on Paired samples t-test. Data are presented as the mean difference (95% CI)

^b^p based on Independent samples t-test. Data are presented as the mean difference (95% CI)

^c^p based on ANCOVA adjusted for baseline values, duration of diabetes, changes in energy intake and BMI. Data are presented as the mean difference (95% CI)

^d^p based on Wilcoxon signed-rank test. Data are presented as the median difference

^e^p based on Mann–Whitney U test. Data are presented as the median difference

^f^p based on Quantile regression adjusted for baseline values, duration of diabetes, changes in energy intake and BMI. Data are presented as the mean difference (95% CI)

## Discussion

The findings of this trial revealed that the supplementation with 3 g/day l-CIT for eight weeks significantly increased the serum levels of CIT and reduced fasting blood glucose concentrations, HbA1c without any significant effects on QUICKI and HOMA-B, serum IL-6, MCP-1, and TLR-4 concentrations compared to the placebo in the patients with T2D.

We found that the l-CIT supplementation significantly increased the serum levels of CIT, which is in line with some human and animal studies [33–35]. It was also observed that fasting blood glucose concentration and HbA1c significantly decreased without any change in HOMA-B and QUICKI. Several studies suggested the hypoglycemic effects of l-CIT in animals. For example, the supplementation with watermelon (skin, rind, flesh) in obese male mice resulted in a significant decrease in fasting blood glucose and insulin resistance using HOMA-IR in the watermelon rind group [36]. Moreover, CIT supplementation for eight weeks in obese rats [37], for 11 weeks in high fat (HF) diet-fed mice [26], as well as in other animal studies [27, 38, 39] after the supplementation with various components of watermelon resulted in a reduction in fasting blood glucose levels, improved insulin resistance and increased insulin sensitivity**.** However, Romero et al. [40] failed to indicate any change in blood glucose levels after the administration of 50 mg/kg/day CIT in type 1 diabetic mice. These findings indicated that l-CIT may have a beneficial effect on improving IR. HbA1c, HOMA-B, and QUICKI were not assessed in the previous studies. The differences in the results could be attributed to the methodology, study duration, l-CIT dose, as well as the sources of l-CIT used in studies. One of the underlying mechanisms of improving glucose homeostasis by l-CIT could be explained by its ability to convert into arginine which can directly act on β cells to stimulate insulin secretion [41, 42]. On the other hand, NO production enhances due to the increases in the arginine bioavailability [43]. Lower levels of NO may reduce glucose transport, resulting in the insulin resistance and diabetes [44].NO is involved in increasing insulin sensitivity in a number of ways, including by increasing the expression and translocation of glucose transporter type 4 (GLUT4) in the skeletal muscle of type 2 diabetic rats [45, 46], as well as by decreasing gluconeogenesis [47] and increasing Hepatic insulin sensitization (HISS) secretion [48]. In addition, suppressing the overactivation of adipocyte c-Jun NH2-terminal kinase (JNK) by inducing its opposing phosphatase MKP-5 (MAPK phosphatase-5) via sGC-cGMP-PKG (soluble guanylate cycles—cyclic guanosine monophosphate production- protein kinase G) signaling pathway, is suggested as another mechanism for NO action which might be expected to improve systemic insulin sensitivity and glucose uptake by preventing excessive postprandial release of saturated fatty acids [49, 50].

In our study, serum IL-6 levels did not significantly decrease after the l-CIT supplementation. However, the serum levels of this inflammatory biomarker were significantly reduced in the l-CIT group. Some in-vitro and in-vivo studies indicated that the l-CIT supplementation reduces serum inflammatory cytokines such as IL-6 [51–53]. Furthermore, a clinical study has supported the IL-6 lowering effect of CIT (10 g/day) for ten days [54]; while, Breuillard et al. [55] reported a positive relationship between IL-6 productions and CIT concentration associated to a decrease in tumor necrosis factor-alpha (TNF-α) production in the peritoneal macrophages in obese diabetic rats. Different factors of transcription for producing these cytokine**,** may be the reason for this discrepancy.

l-CIT supplementation had no significant effect on serum MCP-1and TLR-4 levels, whereas the serum TLR-4 levels were significantly reduced in the l-CIT group compared to the baseline.To the best of our knowledge, we could not find a randomized controlled trial assessing meta-inflammatory biomarkers in T2D patients. However, the effects of the l-CIT or watermelon component, as a source of l-CIT, on TLR-4 and MCP-1 were studied in some animal studies [24, 36, 51, 56]. For example, Jegatheesan et al. [24] reported that the administration of 1 g/kg/d l-CIT in nonalcoholic fatty liver disease (NAFLD) rats reduced TLR4 gene expression. Moreover, in a study on HF-diet-fed mice, a significant drop in MCP-1 serum levels was observed after a 10-week supplementation with watermelon skin powder [36]. As previously mentioned, in T2D and meta-inflammation conditions, elevated FFA levels lead to TLR-4 activation [57], as well as several signal transduction cascades, consequently, the activation of NF-ҠB dimer and expressions of specific target genes in the nucleus [58], such as MCP-1, IL-6, and other pro-inflammatory cytokines, resulted in IR [57]. Reduction in the expression of nuclear factor (NF-ҠB) p65 subunit (RelA), an important transcription factor (TF) involved in the inflammatory response, is a unique mechanism by which l-CIT reduces pro-inflammatory cytokines [58]. Furthermore, NO produced by l-CIT supplementation has the anti-inflammatory properties i.e. NO reduces NF-ҠB activation through *S*-nitrosylation of p50 subunit [59]. A reduction in oxidative stress due to the activation of superoxide dismutase (SOD) and reduced malondialdehyde levels is another anti-inflammatory effects of CIT [60]. The activation of extracellular signal-regulated kinases 1 and 2 (ERK1/2) is reduced in the presence of SOD, which prevent NF-ҠB activation [61]. In this study, factors such as relatively low sample size, low dosage, and short intervention duration might be attributed to unobserved significant changes in serum IL-6 and TLR-4 levels despite their intra-group changes.

A significant increase in serum IL-6 concentration was observed in the placebo group at the end of study. It seems not to be the placebo effect which may be related to the progressive nature of diabetes because previous studies showed the neutral and safe effects of placebo [62].

Furthermore, we found that l-CIT supplementation decreased blood pressure significantly in the l-CIT group, while among group analyses showed no statistically significant difference. Although several studies showed that l-CIT has potentially positive effects to lower the blood pressure or on either SBP or DBP [30, 63–65], there is an evidence indicating that l-CIT does not affect blood pressure [34, 66]. Elevated NO levels through l-CIT supplementation cause the vasodilation of arteries and regulation of BP by stimulating sGC and subsequently, increasing cGMP [67].

Although this study controlled most potential confounders, the relatively small sample size and short-term supplementation could be noted as the limitations of current research.

It is concluded that l-CIT supplementation for eight weeks in the patients with T2D could increase serum levels of CIT and reduce the serum concentrations of fasting blood glucose, HbA1c without any statistically significant effect on insulin sensitivity indices, as well as serum MCP-1, TLR-4, and IL-6 concentrations. Further studies with longer duration are required to investigate the effects of l-CIT on meta-inflammatory biomarkers.

## Data Availability

The datasets used and/or analyzed during the current study are available from the corresponding author upon reasonable request.

## Abbreviations

ADA: American diabetes association
Akt: Protein kinase B
ANCOVA: Analysis of covariance
BMI: Body mass index
CI: Confidence interval
l-CIT: l-citrulline
cGMP: Cyclic guanosine monophosphate
DBP: Diastolic blood pressure
DPP-4 inhibitors: Dipeptidyl peptidase-4 inhibitors
ELISA: Enzyme-linked immunosorbent assay
ERK1/2: Extracellular signal-regulated protein kinases 1 and 2
FFA: Free fatty acid
GLP-1 analogues: Glucagon-like peptide 1 receptor analogues
GLUT4: Glucose transporter type 4
HbA1c: Glycated hemoglobin
HF: High fat
HISS: Hepatic insulin sensitizing substance
HOMA-B: Homeostatic model assessment of β-cell function (HOMA-B) index
HOMA-IR: Homeostatic model assessment of insulin resistance
IDF: International diabetes federation
IL-6: Interleukin 6
IRS1: Insulin receptor substrate 1
MCP-1: Monocyte chemoattractant protein 1
NAFLD: Nonalcoholic fatty liver disease
NF-kB: Nuclear factor kappa B
NO: Nitric oxide
NSAIDs: Nonsteroidal anti-inflammatory medications
PCOS: Polycystic ovary syndrome
PI3K: Phosphoinositide 3-kinase
PRRs: Pattern-recognition receptors
QUICKI: Quantitative insulin sensitivity check index
RAS: Random allocation software
RelA: Nuclear factor (NF-ҡB) p65 subunit
SBP: Systolic blood pressure
SD: Standard deviation
SGLT2 inhibitors: Sodium–glucose cotransporter-2 inhibitors
SOD: Superoxide dismutase
sGC: Soluble guanylate cyclase
TLR4: Toll-like receptor 4
TLRs: Toll-like receptors
TNF-α: Tumor necrosis factor alpha
